# Evaluation of Community and Organizational Characteristics of Smoke-free Ordinance Campaigns in 15 Wisconsin Cities

**Published:** 2005-06-15

**Authors:** David Ahrens, Paul Uebelher, Patrick L Remington

**Affiliations:** Wisconsin Public Health and Health Policy Institute; Wisconsin Public Health and Health Policy Institute, University of Wisconsin Medical School, Madison, Wis; Wisconsin Public Health and Health Policy Institute, University of Wisconsin Medical School, Madison, Wis

## Abstract

**Introduction:**

Smoke-free restaurant ordinance campaigns were conducted in 15 Wisconsin cities during 1992 through 2002. Community and health coalition organizational characteristics varied with each campaign; nine campaigns were successful in enacting ordinances, and six campaigns failed.

**Methods:**

Data on community and coalition characteristics were analyzed. Community characteristics included adjusted gross income, percentage of Democratic voters in recent elections, and county smoking prevalence. Coalition characteristics included the number of supporters identified, leadership experience, level of print news media coverage, and editorial position of local newspaper.

**Results:**

Successful campaigns were more likely to have leadership with high levels of political experience; eight of nine successful campaigns had leadership with high levels of experience, and two of six unsuccessful campaigns had leadership with high levels of experience. Every successful campaign had high levels of newspaper coverage and strong editorial support. None of the unsuccessful campaigns had high levels of news coverage or strong editorial support.

**Conclusion:**

Characteristics controlled or influenced by coalitions are associated with successful outcomes. Community characteristics were not associated with outcomes. These results should assist communities planning to implement smoke-free ordinances or other health policy campaigns.

## Introduction

The purpose of this research was to investigate whether the health coalitions and communities in Wisconsin that were successful in enacting smoke-free restaurant ordinances had characteristics that were different from health coalitions and communities that failed to enact these ordinances.

Since the early 1980s, increasing concern about the health risks of exposure to secondhand smoke has led to a national movement to make public places smoke-free. From the federal law in 1988 that banned smoking on flights of less than 2 hours to the Florida state referendum in 2003 that banned smoking in all workplaces, smoke-free public places have increasingly become the norm. Studies on smoke-free policies in workplaces and public places have shown that such measures are effective in reducing cigarette consumption (1).

As of 2004, more than 1700 U.S. communities had restricted smoking in public places such as restaurants; such ordinances have increased by more than 20% since 1998 (2). Statewide bans have increased most dramatically. In 1998, California was the only state to ban smoking in public places (3). Since then, 10 more states have banned smoking in all workplaces, including restaurants, bars, or both (2).

Changes in state and local laws governing indoor smoking and other controversial public health measures often occur as a result of well-organized efforts of coalitions composed of organizations and individual volunteers. The development and characteristics of these coalitions have been previously examined (4). Organizational approaches of coalitions to the challenge of adopting tobacco-control policies have been examined (5). Previous studies have also examined characteristics of specific communities as well as specific campaign tactics. A study of Massachusetts communities that approved smoke-free restaurant ordinances found that communities with higher educational attainment, higher per capita income, and location in the Boston region were more likely to adopt stronger ordinances (6). Earlier, Bartosch and Pope, also analyzing adoption of smoke-free restaurant ordinances in Massachusetts communities, found that state funding of local tobacco-control boards to support ordinance efforts was the primary indicator of successful enactment (7). Many campaign methods, such as recruitment of campaign staff and identification of supporters, are well documented in the political science literature (8) as well as in the political campaign management literature (9).

From 1992 through 1997, three cities in Wisconsin attempted to pass ordinances requiring smoke-free restaurants. Only one of those three efforts — in the city of Madison during 1992 — was successful. (In addition to these three campaigns, two smaller suburban cities adjacent to Madison also subsequently enacted smoke-free restaurant ordinances: Shorewood Hills in 1992 and Middleton in 1993.) During 1996 and 1997, efforts to pass ordinances by local health advocates in the two other cities were opposed by the local affiliates of the state restaurant association and defeated.

In response to the lack of progress, in 1998 SmokeFree Wisconsin (SFW), a statewide organization funded primarily by The Robert Wood Johnson Foundation and its partner organizations the American Cancer Society, the American Heart Association, and the American Lung Association, developed a new approach to providing technical assistance to local communities interested in passing clean indoor air ordinances. Ordinance proposals were developed; they focused on banning smoking in restaurants with alcohol sales of less than either 33% or 50% of overall revenue. With the exception of relatively minor provisions, such as exemptions of separately ventilated rooms, all of the ordinance proposals were identical.

SFW reviewed the experiences of communities with previously unsuccessful smoke-free restaurant campaigns and found that the individuals and small groups who initiated the campaigns generally lacked the experience and resources necessary to counteract tobacco and restaurant industry opposition. These campaigns may have generated worthwhile activity and strengthened local initiatives, but faulty decisions or lack of early campaign infrastructure led to failure and forced the more experienced supporting organizations such as SFW into "running after a train after it had left the station."

To improve the likelihood of future success, SFW worked closely with local coalitions to develop smoke-free restaurant ordinance campaigns and supported them with dedicated personnel and resources. A manager with experience in political campaigns at the local and state levels was assigned to provide oversight and general technical assistance. The manager identified local on-site organizers with experience in political campaigns or a thorough knowledge of the city's political organization. Many of the same methods used in political campaigns were employed in the ordinance efforts. Although political campaigns focus on identifying and turning out voters for candidates, smoke-free ordinance campaigns identify and turn out voters to contact and influence city council members who vote on ordinances and, in some instances, to participate as volunteers in ordinance campaign activities.

In 1998, SFW selected four cities as likely sites for clean indoor air campaigns: Fond du Lac, La Crosse, Marshfield, and Neenah. The selection was based on the characteristics of successful political campaigns, such as a well-established coalition organization and a large number of identified supporters. Selection of cities was also based on a coalition's experience in other tobacco control issues, such as restricting access of minors to tobacco. After selection of the four cities, several additional municipalities independently began ordinance efforts. The cities of Kenosha and Janesville are notable among these because their economies are largely based on auto assembly plants and because they have large populations of working-class smokers. Kenosha was unique among the cities engaged in ordinance campaigns because when its ordinance passed, none of the city's 150 restaurants was smoke-free.

Five additional cities — Eau Claire, West Bend, River Falls, Ashland, and Oshkosh — initiated ordinance campaigns without the assistance of SFW and were assisted by one or more of the advocacy organizations, such as the American Cancer Society.

## Methods

This study evaluates the campaign and community characteristics of 15 cities engaged in smoke-free restaurant ordinance campaigns from 1992 through 2002 and relates those characteristics to campaign outcomes. It also examines the effectiveness of applying standard political campaign methods to public health policy issues.

As of March 1, 2004, there were 11 smoke-free cities and three smoke-free villages in Wisconsin. Four of these communities were excluded from this study because they are smaller suburbs of either Madison or La Crosse. Also, the city of Wauwatosa was excluded because it passed an ordinance that has been delayed for 2 years. The suburban communities outside Madison and La Crosse were not included in the analysis because the primary reason for the passage of their ordinances was their proximity to cities with well-covered campaigns.

We hypothesized that coalitions with more experienced leaders (10), a larger number of ordinance supporters (11), greater news media coverage, and stronger editorial support (12,13) would be more successful. We also hypothesized that cities with lower average smoking prevalence (14), higher percentages of Democratic voters, and wealthier residents were more likely to pass ordinances. A description of the characteristics examined and their indicators follows.

### Community characteristics

#### Adjusted gross income

Adjusted gross income is a standard measure of community wealth (15). We hypothesized that wealthier communities would be more likely to support clean indoor air ordinances because they may have a lower percentage of smokers (14,15). Wealthier communities, however, may also be more likely to be politically conservative and thus less likely to support the regulation of businesses and individual behavior.

#### Democratic voting percentage

Political preference was measured by the percentage of voters who voted for the Democratic candidate at the "top of the ticket" (e.g., governor, senator, or president) in each jurisdiction in three statewide and national elections (1998,1998, 2000, and 2002) as identified by state election board records (16). We hypothesized that cities with a higher Democratic electorate would be less opposed to regulation and thus more likely to support ordinances.

#### Smoking prevalence

Smoking prevalence was estimated at the county level by analyzing data from the 2002 Wisconsin Smoking-Attributable Mortality, Morbidity and Economic Costs report (17). We hypothesized that communities with high smoking prevalence were less likely to support the ordinance because of greater opposition from smokers and businesses that cater to large numbers of smokers.

### Campaign characteristics

#### Coalition leadership experience

Coalition experience was considered high if leadership had extensive experience in policy change at the municipal level, extensive experience in political organization (e.g., campaign management), or extensive civic involvement that included working with a local city council. Coalition experience was considered low if leadership did not have such experience. Two authors separately reviewed factors related to each coalition and rated experience as high or low. Raters had a high level of agreement on ratings. We hypothesized that coalitions with more experienced leadership would have greater success.

#### Numbers of supporters identified

Identification and mobilization of potential supporters in the community was critical to success because supporters influence primary decision makers — members of the city council. The process of influencing city council members occurred primarily through individual telephone calls from constituents to council members, but the process also included e-mails, letters, and personal visits. The most common methods for identifying and mobilizing supporters were telephone calls (i.e., cold calls) and newspaper inserts. Both methods solicited support and asked if the respondent was affected by secondhand smoke. Cold calls were made to individuals listed in public voter files. Newspaper inserts consisted of postage-paid postcards inserted into daily newspapers. Postcards were returned to the coalition. A secondary source of potential supporters was generated through petitions gathered by coalition volunteers. The effectiveness of petitions was often limited by the difficulty of reading names and addresses of signers. Data from all methods were entered into a central local database, and the database was used to produce mailing lists.

We hypothesized that coalitions with the highest percentage of supporters, determined as a percentage of the total number of voters in the most recent gubernatorial election, would have greater success in enacting a smoke-free ordinance.

#### Level of newspaper coverage

Newspaper coverage of the ordinance story was categorized as high, moderate, or light. Coverage was considered high when the ordinance campaign was covered throughout the campaign and included coverage on the front page of the local newspaper. This level included news stories focusing on health and economic concerns. Moderate coverage consisted of occasional articles on citizen views (primarily views of restaurant owners) and major events related to the ordinance campaign. News media coverage limited to the final stages of the decision-making process was considered light. Two authors and one or two individuals from each coalition rated the coverage level. All newspaper coverage was monitored. We hypothesized that campaigns receiving high levels of coverage were more likely to have the ordinance passed.

#### Editorial position of the major newspaper

The authors and one of the local coalition leaders collected and analyzed editorials on the campaign and the proposed ordinance and categorized the editorials as supportive or oppositional. We hypothesized that campaigns receiving supportive editorial coverage would be more likely to gain passage of their ordinance.

A panel of three participant-observers made independent qualitative assessments of the characteristics related to coalition leadership experience, extent of print news media coverage, and editorial positions. Raters were highly familiar with each campaign under their observation and used a questionnaire with scales ranging from high to low. Because of the small sample size, we did not test interrater reliability. There was approximately 90% agreement on the 45 measures among panel members in their assessment of each characteristic. We used *t* tests and the Fisher exact test to assess statistical significance.

## Results

Nine of the 15 campaigns examined in this study were successful in enacting smoke-free ordinances. Table 1 summarizes the community and coalition characteristics of the cities. Compared with cities with unsuccessful campaigns, cities with successful ordinance campaigns had slightly higher adjusted gross income ($37,782 vs $36,437); a greater percentage of Democratic voters (56% vs 49%); and slightly higher smoking rates (25.7% vs 23.3%) (Table 2).

The leadership of coalitions involved in successful campaigns had more experience (with eight of nine having high levels of experience) than leadership of unsuccessful campaigns (with two of six having high levels of experience). In addition, successful coalitions had more identified supporters: an average of 13% of the population voting in the most recent gubernatorial election was identified as supporters of the successful campaigns, compared with an average 9% of the population identified for the unsuccessful campaigns. Excluding one coalition that did not systematically identify supporters, successful coalitions identified between 6% and 24% of the population that had voted in the previous gubernatorial campaign. Although the difference between the percentage of identified supporters in successful and unsuccessful campaigns was not statistically significant, this finding should not be interpreted to mean that this strategy is not essential to a successful outcome.

All of the communities that passed an ordinance had high levels of coverage of the ordinance campaign in the print news media. With the exception of one community (River Falls) in which the newspaper did not take a position and another (Madison) that had two daily papers with opposing positions, all of the communities that passed an ordinance also had the editorial support of their local newspaper. In contrast, local newspapers did not provide editorial support to any of the unsuccessful campaigns.

## Discussion

In this 15-city evaluation study, success in enacting smoke-free ordinance campaigns was related to three key characteristics. First, coalitions that had leadership with high levels of prior political experience tended to be more successful. Researchers have examined the characteristics of successful coalitions (4-6). Coalitions with politically experienced paid and volunteer leadership have the internal and external resources to organize campaigns with the capacity to overcome opposition from the restaurant and tobacco industry. Highly functioning and experienced coalition leadership is also able to operate successfully in a highly conflictive political environment. The coalitions and coordinators in successful communities knew policy makers and council members and had experience in news media relations. Groups with minimal experience in the policy-making process have difficulty learning the fundamentals of policy in the context of a highly controversial proposal. Many groups that initiate smoke-free ordinances underestimate the political strength of the opposition (18).

Second, positive editorial support and a high level of print news media coverage were strongly associated with success. It is unknown, however, whether the supportive editorial position of the newspaper, activities of the coalition, or opponents of the ordinance modulate levels of coverage. Coalitions were generally unable to overcome opposition by local newspapers. Conversely, coalitions with limited policy or campaign experience were still able to win ordinances with strong support and extensive coverage from local newspapers. Although this should not discourage organizations from pursuing ordinances in communities with oppositional news media, it should highlight the importance of early and sustained attention to this important opinion-shaping factor.

Third, print news media coverage was much more extensive in cities with successful campaigns. The news media plays an important role in setting agendas, defining issues, legitimizing issues of public concern, and influencing the public, especially policy makers (19,20). Coalitions have the ability to influence the news media through attention-worthy events, organizational relationships, and editorial influence (12). Local newspaper opposition to smoke-free restaurant ordinances and negative editorials played a central role in the defeat of proposals.

While editorials marginally influence the public, they are a powerful influence on decision makers and opinion leaders. Many politicians will not support proposals that are strongly opposed by the press (13). Conversely, they are encouraged to support proposals and take risks when their position is supported in the news media (21).

In addition to the importance of strong editorial support is the related positive factor of extensive news media coverage. News media coverage may have a legitimizing effect and increase the level of information on secondhand smoke available to the community.

Communities that enacted clean indoor air measures were only marginally wealthier than those that did not. Although economic status is closely related to smoking status, it does not appear to influence preference for an ordinance at the community level. Despite the association between higher economic class and lower smoking rates, political conservatism in wealthier communities may have militated against acceptance of the ordinance because of the perception that the ordinance would unnecessarily regulate individual behavior.

In our study, coalitions that identified a significant percentage of the electorate as supporters of the ordinance and encouraged them to contact their elected officials were slightly more likely to gain passage of the ordinance. Identification and activation of supporters appears to be important to successful campaigns. With a single exception of Madison, communities that did not identify large numbers of supporters were often unable to overcome opposition organized by the restaurant industry.

Coalitions with a relatively large number of mail- or telephone-identified supporters were able to generate numerous contacts with city council members. Although our small study did not demonstrate a strong association, widely accepted methods of political campaign management hold that higher levels of getting out the vote, or GOTV, are more likely to produce electoral victories (11). Our study did not disprove that hypothesis.

Our results indicate that cities with successful ordinance campaigns had a somewhat higher proportion of Democratic voters. This association was not, however, consistent. Two of the nine successful ordinances were passed in predominantly Republican cities, whereas three of the six failed campaigns occurred in predominantly Republican cities. This tendency toward passage of ordinances in Democratic cities may result from greater acceptance of governmental regulation of private businesses and individual behavior.

Finally, we observed slightly higher smoking prevalence in communities that passed an ordinance. Municipalities that passed ordinances were located in counties that ranged from the lowest to the highest estimated smoking prevalence rates in the state. Municipalities that failed to pass ordinances also had a wide range of prevalence rates.

The results of our study confirm the findings of some researchers, diverge from others, and support continued study in other areas of community interventions. Confirming the findings of Blaine et al, we found that well-organized tobacco control coalitions with experienced leaders had advantages in securing policy change (5). Our results also confirmed the importance of identifying supporters: we found a discernible, though not statistically significant, difference between numbers of supporters in communities that were and were not successful in enacting ordinances. This observation confirms the findings of research on the importance of identifying and mobilizing supporters in political campaigns (11). The impact of mobilization of the affected population as active supporters of a community health action is a worthy topic of continued study.

We did not, however, find differences in outcomes stemming from community characteristics such as high income, as did Skeer et al in their study of Boston area ordinance campaigns (6). Finally, we found that both the theory and practice of media advocacy was critically important to successful passage of the ordinance (12,21).

Several limitations should be noted in this study. First, the small number of communities, most of which were predominantly white, may limit the generalizability of the findings. Research indicates that cities with significant communities of color may have higher levels of support and acceptance of smoke-free ordinances than communities with predominantly white populations (22). Also, two of the three authors rated coalition experience. A larger group might have arrived at a different result. Finally, as with all observational research, this is a small sample with no random assignment to different campaign strategies.

This study of smoke-free ordinance campaigns in 15 Wisconsin municipalities identifies characteristics of successful campaigns. Successful campaigns were organized by coalitions that were more experienced, identified higher numbers of citizen supporters, and encouraged more supporters to contact policy makers. Campaigns were also more successful when there were high levels of news media coverage and editorial support from local newspapers. Increasingly, public health practitioners recognize the importance of policy as an effective means of creating a more healthful environment (23). Our results should assist communities planning to implement smoke-free ordinances or other health policy campaigns.

## Figures and Tables

**Table 1:** Characteristics of Communities and Smoke-free Coalitions in 15 Wisconsin Cities, 1992–2002

**City** **(population)**	**Adjusted Gross Income** **(Mean, $)**	**Democratic Voters** **(%)**	**County Smoking Prev-** **alence^a^** ** (%)**	**Voters Identified as Sup-** **porters^b^** ** (%)**	**Level of Coalition Leadership Exper-** **ience^c^**	**Level of Newspaper Coverage^c^**	**Editorial Position of Local News-** **paper(s)**

**Unsuccessful campaigns**							

Beloit (35,775)	29,455	54	22	0	L	M	Oppose
Marshfield (18,800)	38,727	44	24	10	L	L	Oppose
Oshkosh (64,132)	36,125	47	28	10	H	M	Oppose
Sheboygan (50,792)	34,683	52	20	0	L	M	Oppose
West Bend (28,630)	41,788	39	22	11	L	M	Oppose
Stevens Point (24,551)	37,847	60	NA	22	H	M	d

**Successful campaigns**							

Ashland (8,651)	27,745	59	NA	16	L	H	Support
Eau Claire (63,214)	37,607	56	23	6	H	H	Support
Fond du Lac (42,619)	32,703	44	27	14	H	H	Support
Kenosha (91,853)	36,903	63	36	18	H	H	Support
Janesville (60,775)	40,644	62	22	10	H	H	Support
La Crosse (51,781)	35,141	56	24	8	H	H	Support
Madison (195,432)	43,323	68	20	0	H	H	d
Neenah (24,687)	43,235	43	28	24	H	H	Support
River Falls (12,811)	42,736	54	NA	19	H	H	d

a Smoking prevalence rates are reported for county in which city is located. NA indicates that prevalence rate is not available for county.

b Supporters were identified through telephone or mail surveys as a percentage of voters in most recent gubernatorial election.

c H indicates high level of support; M, medium level of support; and L, low level of support.

d Either no editorial position taken or opposing position between two newspapers.

**Table 2:** Characteristics of Successful (Passed) and Unsuccessful (Did Not Pass) Smoke-free Campaigns in 15 Wisconsin Cities, 1992–2002

**Characteristic**	**Ordinance Passed** **(N=9)**	**Ordinance Did Not Pass** **(N=6)**	***P***
Adjusted gross income (mean, $)	37,782	36,437	.61
Democratic voters (mean, %)	56	49	.13
County smoking prevalence (mean, %)	25.7	23.3	.37
Voters identified as supporters (mean, %)	13	9	.35
High level of coalition leadership experience	8/9	2/6	.047
High level of newspaper coverage	9/9	0/6	<.001
Newspaper editorial support^a^	7/7	0/5	.001

a Analysis was limited to newspapers that expressed an editorial opinion.
